# The efficacy of hyperthermia-based multimodal therapy is dependent on gatekeeper protein, BID

**DOI:** 10.1080/02656736.2025.2544017

**Published:** 2025-08-11

**Authors:** Sarah Helmueller, Xinxin Song, Dong-Hyun Kim, Yong J. Lee

**Affiliations:** a Department of Biomedical Sciences, Cedars-Sinai Medical Center, Los Angeles, CA, USA; b Department of Surgery, UT Southwestern Medical Center, Dallas, TX, USA; c Department of Radiology, Feinberg School of Medicine, Northwestern University, Chicago, IL, USA; d Robert H. Lurie Comprehensive Cancer Center, Northwestern University, Chicago, IL, USA; e Department of Biomedical Engineering, University of Illinois at Chicago, Chicago, IL, USA; f Department of Biomedical Engineering, McCormick School of Engineering, Northwestern University, Evanston, IL, USA

**Keywords:** Ferroptosis, apoptosis, crosstalk, BID, bak, bax

## Abstract

**Objectives:**

The combination of ferroptotic agent artesunate (ART) and apoptotic agent rhTRAIL (recombinant human tumor necrosis factor-related apoptosis-inducing ligand) has been shown to synergistically enhance apoptosis in various cancer cell lines via crosstalk between the endoplasmic reticulum (ER) stress response, the rhTRAIL-induced extrinsic cell death receptor pathway, and the intrinsic BID-Bax-mitochondrial-dependent-apoptosis pathway. This synergistic interaction has been demonstrated to be effective in multiple types of cancer cell lines, making artesunate combined with rhTRAIL a promising second-line therapy for colon cancer patients after cytoreductive surgery and chemotherapeutic treatments. To further enhance the second-line therapy’s tumoricidal effect, a multimodal therapy was developed by combining artesunate, rhTRAIL, and hyperthermic conditions where samples were treated at 42°C for 1h.

**Methods:**

The effects of this therapy were tested in human colon carcinoma HCT116 and pancreatic adenocarcinoma BxPC-3 cell models. The cytotoxic and synergistic effects were analyzed using fluorescence microscopy, cell survival assays, and protein analysis through Western blotting.

**Results:**

Our findings demonstrated a significant enhancement of apoptosis when artesunate and rhTRAIL treatments were combined with heat exposure. The synergistic and apoptotic effect of the agents was effectively abrogated in BID-deficient and BID mutant-type cells as well as Bax-deficient cells, but not Bak-deficient cells.

**Conclusions:**

The results suggest that BID acts as a key gatekeeper molecule of apoptosis during hyperthermia-based multimodal treatment. These findings raise important questions about the underlying mechanisms of heat-induced apoptosis and its involvement in orchestrating various cellular stress pathways.

## Introduction

Colorectal peritoneal carcinomatosis (CPC) is the second most prevalent cancerous disease to affect the peritoneal cavity following colorectal cancer and is regarded as a lethal condition with a poor prognosis [1,2]. In the past, CPC was considered a terminal disease stage for which palliative treatment was the only option. However, hyperthermia has been successfully used alongside chemotherapy in CPC patients after cytoreductive surgery (CRS) [1–9]. Heat treatment, applied between 40 and 44°C for 1–2 h, induces cellular stress *in vivo*, effectively targeting tumor environments due to their distinct physiological features, such as low pH and hypoxia [9,10]. Additionally, heat treatments can enhance cytotoxic and reactive oxygen species (ROS) activity, making cancer cells more vulnerable to second-line therapies with minimal harm to normal cells [7–9,11]. In the clinic, local hyperthermia methods, such as Hyperthermic Intraperitoneal Chemotherapy (HIPEC), are commonly used for abdominal and colon cancers, where heated chemotherapy is pumped into the targeted area [7]. Clinical trials indicate that combining hyperthermia with radiation and chemotherapy yields better outcomes than any single treatment alone [7–9,11]. However, while CRS combined with HIPEC has improved response rates, complete recoveries remain rare, and cancer recurrence is still common [12]. Therefore, an improved strategy is needed to enhance the efficacy of CRS and HIPEC.

Building on the successes of CRS and HIPEC, we explored a hyperthermia-based multimodal therapy (rhTRAIL + artesunate) as a second-line treatment to eliminate microscopic tumors after CRS and reduce chances of cancer recurrence. Our previous research demonstrated the synergistic effects of artesunate (ART) and rhTRAIL (tumor necrosis factor-related apoptosis-inducing ligand) in enhancing apoptosis in cancer cell lines [13–15]. rhTRAIL induces apoptosis by binding to death receptors 4 and 5 (DR4 and DR5) on cancer cells without affecting normal cells *in vivo* [16,17]. Artesunate, an anti-malarial drug, acts as a strong ferroptotic agent [13,18,19] as well as a weak apoptotic agent [20,21], generating ROS and inducing irondependent lipid peroxidation, which activates the endoplasmic reticulum (ER) stress response and promotes mitochondrial-dependent apoptosis [22–25]. In our studies, ART enhanced rhTRAIL-induced apoptosis through crosstalk between the ER-stress-mediated mitochondrial pathway and the BID-Bax-mitochondrial apoptotic pathway [15,23,26]. While we had previously observed enhanced tumoricidal efficacy with the combination of rhTRAIL and hyperthermia, the effect of heating cells while adding ART to rhTRAIL had not been tested [9]. Other studies have highlighted the role of hyperthermia in enhancing rhTRAIL-induced apoptosis in cancer cells through caspase activation and the BID-Bax-mitochondrial-dependent apoptotic pathway [24,25]. Similarly, heat combined with cisplatin has shown synergistic effects in prostate cancer cells, and in another study, Withaferin A enhanced hyperthermia-induced apoptosis [27,28]. Based on our previous findings and published literature, we hypothesized that the synergistic effects of ART and rhTRAIL could be further enhanced under hyperthermic conditions.

In this study, we observed that a hyperthermia-based multimodal treatment with ART and rhTRAIL significantly enhanced apoptosis in cancer cell lines compared to any single treatment. Interestingly, heat combined with ART significantly increased cytotoxicity and cell death, a result not found with ART treatment alone. These findings raised questions about the mechanisms underlying hyperthermia-induced cellular stress and cytotoxicity. First, we investigated apoptosis as the mechanism of cell death during hyperthermia-enhanced multimodal treatment, Then, we explored the roles that heat shock proteins and Caspase-2 play in a hyperthermia-based multimodal treatment. Lastly, we identified that the BID and Bax-associated-mitochondrial-dependent pathway plays a crucial role in regulating the effects of the hyperthermia-based multimodal treatment [28].

## Materials and methods

### Cell lines and cell culture conditions

Human colorectal carcinoma HCT116 cells and human pancreatic cancer BxPC-3 cells were previously obtained from American Type Culture Collection (ATCC, Manassas, VA). BID-deficient (BID^−/−^) HCT116 cells stably expressing the BID D60E or G94E vector (BID^−/−^/D60E or BID^−/−^/G94E) were provided by Dr. Luo (University of Nebraska Medical Center, Omaha, NE). Bak-deficient (Bak^−/−^), Bax-deficient (Bax^−/−^), and Bax/Bak Double Knockout (Bax^−/−^ Bak^−/−^ DKO) HCT116 cells were provided by Dr. B. Vogelstein (Johns Hopkins University, Baltimore, MD). Cell lines were maintained in mediums as listed: HCT116 cells in McCoy’s 5 A and BxPC-3 cells in Rosewell Park Memorial Institute (RPMI)-1640 supplemented with 2mM glutamine. All cell lines were maintained with 10% fetal bovine serum and 1% penicillin streptomycin and incubated in a humidified atmosphere at 5% CO_2_ at 37°C.

### Chemicals and reagents

For production of recombinant human TRAIL (rhTRAIL), a human TRAIL cDNA fragment (amino acids 114–281) obtained by RT-PCR was cloned into a pET-23d plasmid (Novagen, Madison, WI), and His tagged TRAIL protein was purified using the Qiagen express protein purification system (Qiagen, Valencia, CA, USA). Artesunate was purchased from Sigma-Aldrich (Cat#A3731–100MG).

### Cell morphology study

HCT116 and BxPC-3 cells were treated with ART and/or heat and/or rhTRAIL and observed under an ECHO Revolve phase microscope (ECHO, San Diego, CA, USA) in the bright field mode.

### Cell death and viability assay

Cell death was measured using the trypan blue exclusion assay to detect the cells’ plasma membrane integrity. After 20 h of ART treatment, HCT116 cells were treated with rhTRAIL, some were put in a hot water bath (42°C) for 1h, and then in the incubator at 37°C for 3 more hours. To quantify the percentage of cell death, immediately after treatment cells were trypsinized and stained with 0.4% trypan blue, followed by counting using the LUNA^™^ Automated Cell Counter (L10001, Logos Biosystem, Anyang, Gyeonggi-do, South Korea) according to the manufacturer’s instructions. The authors acknowledge the limitations of this method, particularly in distinguishing early membrane permeability from long-term loss of proliferative capacity. However, this method was selected due to its efficiency and feasibility given the multiple treatment conditions, time points, and biological replicates included in the study. The cells were assayed immediately after the multimodal treatment was completed. This allowed us to consistently and rapidly evaluate viability across a broad experimental framework. Continually, given the time-sensitive and comparative nature of this paper’s experiments, this approach allowed us to efficiently assess differential cytotoxicity.

### Western blotting and antibodies

Immunoblotting was carried out as previously described [13]. The following antibodies were used in this study: anti-PARP-1 (#9532), anti-caspase-3 (#9668), anti-caspase-8 (#9746), anti-caspase-9 (#7237), anti-caspase-2 (#2224), anti-HSP70 (#4872), anti-HSP90 (# 4877), anti-Bax (#2772), anti-Bak (#3814), and anti-BID (#2003) (Cell Signaling Technology, Beverly, MA); and anti-actin, goat anti-rabbit IgG-horseradish peroxidase (HRP), and goat anti-mouse IgG-HRP (Santa Cruz Biotechnology, Santa Cruz, CA, USA).

### Statistical analysis

Statistical analysis was performed using one-way and two-way analysis of variance (ANOVA) followed by Sidak’s or Tukey’s multiple comparisons test as indicated using GraphPad Prism 8 software. P-values less than 0.05 were defined as statistically significant. P-values are indicated as follows: *p < 0.05; **p < 0.01; ***p < 0.001. Please reference Table 1 for P-values and significance data.

### Combination Index analysis

Combination index (CI) analysis was performed using CompuSyn software (ComboSyn, Inc., Paramus, NJ, USA). CompuSyn is a reliable and widely used software developed by Dr. Dorothy Chu in 2005 to calculate the Combination Index (CI) for multiple drug treatments, doses, and combinations, therefore, it was an ideal choice for our study investigating multimodal treatments using varying concentrations of ART and rhTRAIL, with or without heat. The software is based on the median-effect principle of the mass-action law and the combination index equation, offering a streamlined approach to pharmacodynamic analysis. The median-effect equation (MEE) quantifies dose-effect relationships using two key parameters: the median-effect dose (Dm), indicating potency, and the slope (m), reflecting the shape of the dose-response curve. The combination index equation (CIE) extends this model to classify drug interactions as synergistic (CI < 1), additive (CI = 1), or antagonistic (CI > 1). Unlike traditional methods requiring extensive data and curve fitting, CompuSyn uses a “Top Down” approach that requires fewer dose points and leverages internal reference parameters for predictive simulations. In our study, the extent of antagonism/synergism was determined using the software described above, to determine each treatment combination’s CI value. A CI value above 1 suggest antagonism between the drugs, whereas CI values below 1 indicate synergy. CI values in the 0.9–1.10 range mainly indicate additive effects, those between 0.9–0.85 suggest slight synergy, those in the range of 0.7–0.3 indicate moderate synergy, and those less than 0.3 suggest strong synergy. Refere Table 2 for summary of CI data.

### Densitometry analysis

Densitometric quantification of Western blot bands was performed using ImageJ analysis software. For each blot, the density profile of individual bands was generated, and peak areas were measured using the gel analysis tool. The integrated density values corresponding to cleaved PARP-1 and full-length PARP-1 were used to calculate their relative ratio. A higher cleaved-to-full-length PARP-1 ratio was interpreted as an indicator of increased apoptotic activity. All densitometric measurements were normalized to corresponding loading controls (e.g. β-actin) by calculating a normalization factor and applying that factor to the PARP-1 data to account for potential variability in protein loading and transfer.

### Annexin V assay

Annexin V detects and binds to a phospholipid that is located on the outer cell membrane in apoptotic cells. HCT116 wild-type and BxPC-3 cells were plated in 12-well cell culture plates. After 20 h of ART treatment, cells were treated with rhTRAIL, some were put in a hot water bath (42°C) for 1h, and then in the incubator at 37°C for 3 more hours. Post-treatment, cells were washed once with PBS. Binding buffer, Propidium-Iodine (PI) stain, and Annexin V reaction mixtures were added to the samples and incubated at 37°C for 20 min in the dark using the Annexin V-FITC/PI Apoptosis Detection Kit (MedChem Express, Cat#HY-K1073), following the manufacturer’s instructions. After incubation, the cells were washed once with PBS. Hoechst dye was then added by diluting the stock solution 1:2000 in PBS, followed by incubation at room temperature for 10 min in the dark. After washing once again with PBS, an ECHO fluorescence microscope was used to capture images. The Annexin V-FITC stains green under a fluorescence microscope, indicating cells in early-apoptosis stages, while PI stain illuminates as red under a fluorescence microscope, indicating cells in late-apoptosis stages.

### TUNEL assay

Detection of apoptosis was done using the terminal deoxynucleotidyl transferase dUTP nick end labeling (TUNEL) method. HCT116 wild-type cells were plated in 12-well plates. After 20 h of ART treatment, cells were treated with rhTRAIL, half of the samples were put in a hot water bath (42°C) for 1h, and then in the incubator at 37°C for 3 more hours. Post-treatment, cells were washed once with phosphate buffered saline (PBS). TUNEL reaction mixture was added to the samples and incubated at 37°C for 1h in the dark using the *In Situ* Cell Death Detection Kit, Fluorescein (Roche, Cat#11684795910), following the manufacturer’s instructions. The cells were then washed once with PBS, Hoechst dye was then added by diluting the stock solution 1:2000 in PBS, followed by incubation at room temperature for 10min in the dark. The cells were then washed once with PBS again and examined under an ECHO fluorescence microscope.

### JC-1 assay

JC-1 dye (mitochondrial membrane potential probe, ThermoFisher Scientific, Cat#T3168) was used to analyze mitochondrial membrane potential (ΔΨm) of cells after the multimodal treatment. HCT116 cells in a 12-well plate were treated for 20h with ART, and then treated with rhTRAIL. Immediately, some samples were put in a heated water bath (42°C) for 1h, and then in the incubator at 37°C for 3 more hours. After treatment, cells were stained with JC-1 dye as per the manufacturer’s instructions. After incubation with JC-1 dye, the cells were washed with PBS. Hoechst dye was then added by diluting the stock solution 1:2000 in PBS, followed by incubation at room temperature for 10min in the dark. After washing again with PBS, an ECHO fluorescence microscope was used to capture images.

### Mean fluorescence intensity analysis

For cytotoxicity analysis using immunofluorescence data, the Mean Fluorescence Intensity (MFI) of images captured during Annexin V, JC-1, TUNEL, and Hoechst staining were calculated using ImageJ software. After using the “Image”<“Color”<”Split Channels” function, the original images were split into RGB channels and analyzed in grayscale. Then, MFI values were measured from three randomly selected fields for each experimental condition. For the JC-1 MFI quantification, JC-1 Red, JC-1 Green, and Hoechst MFIs were quantified using ImageJ software. The JC-1/Hoechst ratio was obtained by dividing either the JC-1 Red or Green MFI by the Hoechst MFI in each field. For both the TUNEL and Annexin V assays, MFI analysis was performed in the same fashion. In Annexin V assay analysis, the MFI of Annexin V (Green Channel - early apoptosis), PI Stain (Red Channel - late apoptosis), and Hoechst staining was quantified using ImageJ, and then the Annexin V/Hoechst ratio or PI/Hoechst ratio were obtained as described and averaged over triplicate samples. For the TUNEL assay, again, MFI of TUNEL and Hoechst stains were quantified using ImageJ software. The TUNEL/Hoechst ratio was obtained by dividing the TUNEL MFI by the Hoechst MFI and the resulting value is the average over three samples.

## Results

### Combination therapy potentiates cytotoxic and apoptotic effects

**Figure 1.**
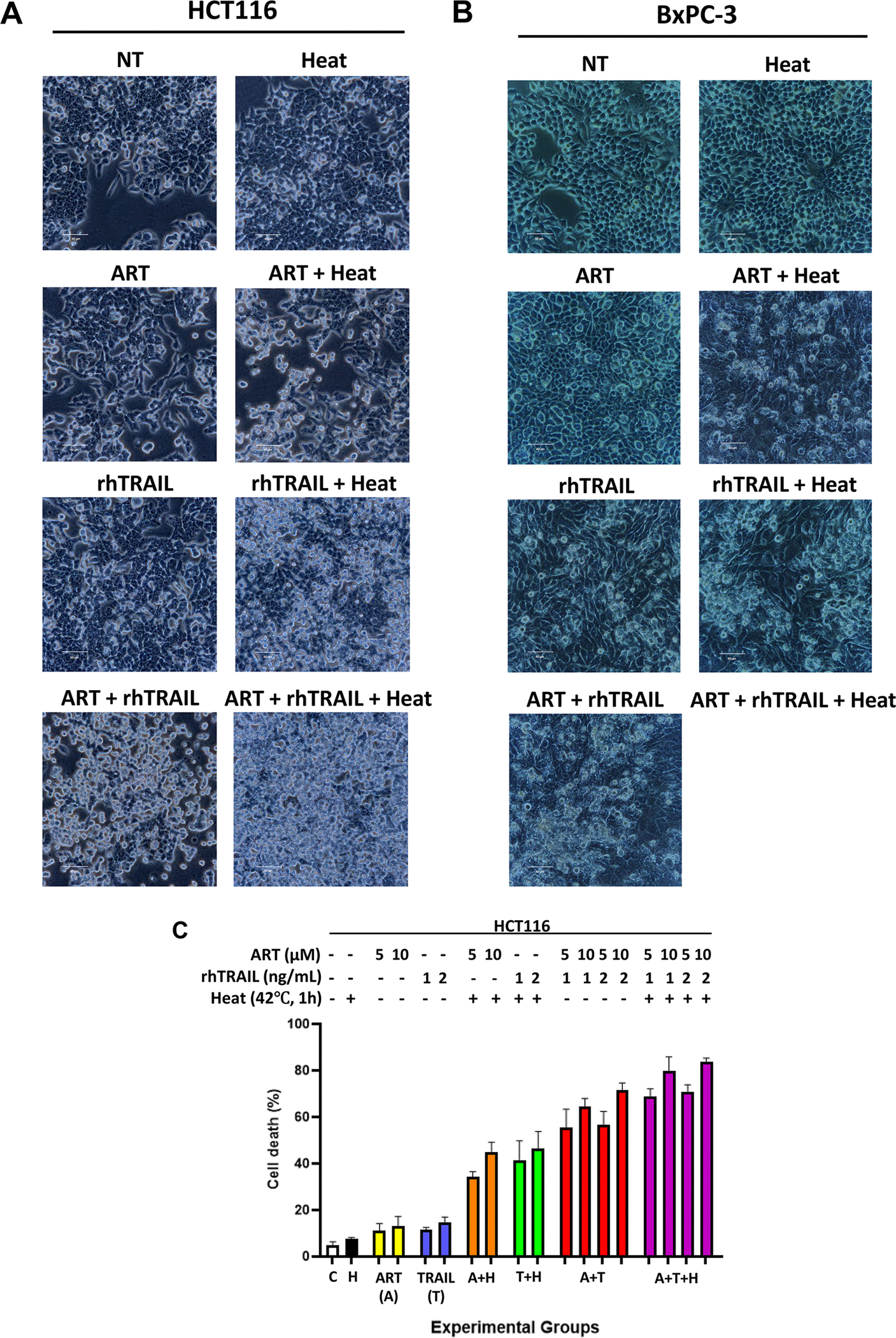

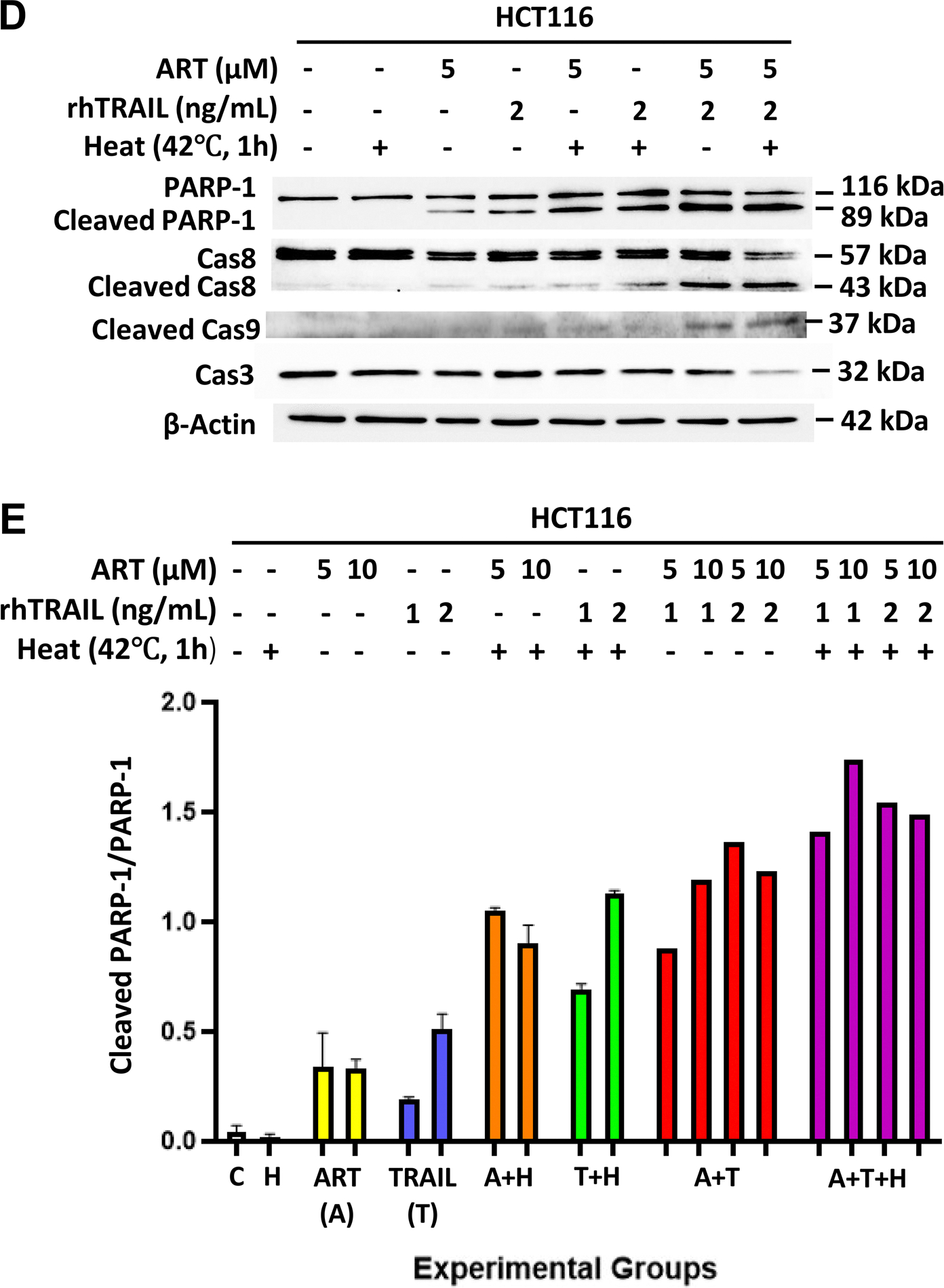
Hyperthermia promotes multimodal (ART +TRAIL) treatment-induced cytotoxicity and apoptosis in human colorectal carcinoma HCT116 cells and pancreatic adenocarcinoma BxPC-3 cells. Cells were treated with 5 or 10μM of artesunate (ART) for 20h at 37°C, and then with or without 1 or 2 ng/mL rhTRAIL for additional 4h. Some samples were subjected to hyperthermia treatment in a water bath at 42°C for 1 h, and then placed back in the 37°C incubator for another 3h. (A, B) After treatment, morphological features of HCT116 and BxPC-3 cells treated with 10μM of ART and/or 2 ng/mL rhTRAIL and/or 42°C were analyzed under an ECHO revolve microscope. (C) After treatment, HCT116 cell death was determined by a trypan blue exclusion assay. Error bars represent the mean ± SD from triplicate experiments. Error bars represent the mean ± SD from triplicate experiments. P-values: *, 0.05; **, 0.01; ***, 0.001 are represented in Table 1 (D) Whole-cell HCT116 extracts were analyzed with an immunoblotting assay using indicated antibodies. (E) Protein expression ratio of cleaved PARP-1 to full length PARP-1 from Western blot in Figure 1D was analyzed using ImageJ software and calculated. Error bars represent the mean ± SD from replicate samples.

To evaluate the effects of hyperthermia on the synergistic interaction between artesunate (ART) and rhTRAIL, we first examined morphological changes in treated human carcinoma HCT116 cells and pancreatic adenocarcinoma BxPC-3 cells. Cells were treated with 5 or 10μM ART, with or without 1 or 2 ng/mL rhTRAIL, and half of the samples were heated at 42°C for 1h. Figure 1(A) (morphology) and 1B (cytotoxicity) shows that heat alone induces minimal effect, but in combination with ART and/or rhTRAIL, it enhances cell death, evident by increased cell blebbing and detachment in both HCT116 and BxPC-3 cells.

To further evaluate the cytotoxic effects of the multimodal treatment, Trypan Blue staining was performed to assess loss of membrane integrity and quantify cell death (Figure 1(C)). In addition, immunoblot analysis was conducted to examine apoptosis by detecting PARP-1 cleavage and caspase cleavage (activation), both hallmark features of apoptotic cell death (Figure 1(D,E)). Cell death percentage and Western Blot results show that ART alone induces minimal cytotoxicity, but when combined with heat, cytotoxicity and PARP-1 cleavage are significantly enhanced. The same effect is observed with rhTRAIL, where the addition of heat increases cytotoxicity and PARP-1 cleavage compared to rhTRAIL alone (Figure 1). Additionally, heat further enhances cytotoxicity and apoptosis in the ART and rhTRAIL combination treatment (Figure 1). The effects of various combination treatments on enhanced cytotoxicity are condensed in Table 1, which summarizes P-values calculated from cell death data presented in Figure 1(C). Hyperthermia significantly increased the cytotoxicity induced by varying concentrations of ART, rhTRAIL, or their combination. However, the extent of enhancement differed depending on the treatment conditions.

Continually, using the survival results from multiple dosage experiments, Combination Index analysis in Table 2 demonstrates moderate to strong synergy in all samples treated with ART and rhTRAIL in combination with heat, as well as ART and rhTRAIL together without heat. The CI values in Table 2 highlight key interactions between ART, rhTRAIL, and hyperthermia. Notably, the dual treatment of ART + rhTRAIL yielded strong synergy (CI < 0.01), indicative of a potent apoptotic response. In contrast, the triple combination with hyperthermia exhibited moderate synergy (CI ~0.5–0.7), suggesting an additive yet beneficial effect.

### During hyperthermia-based multimodal treatment, HCT116 cells respond to heat stress by elevating the level of heat shock proteins and reducing the level of caspase-2

**Figure 2.**
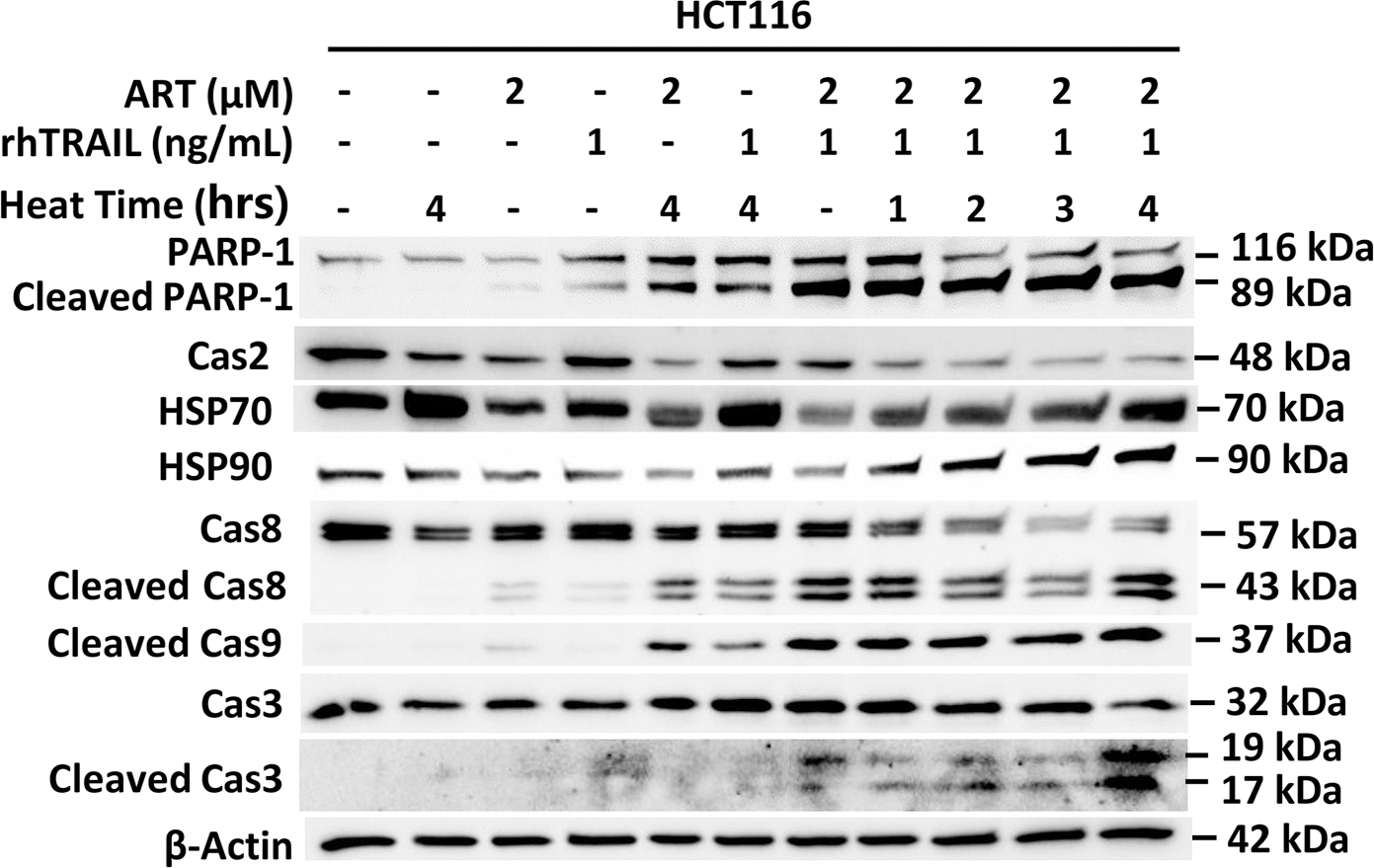
Modulation of caspase-2 and HSP protein levels in hyperthermia-enhanced apoptosis during multimodal treatment. HCT116 cells were treated with 2μM ART for 20h at 37°C, followed by treatment with or without 1 ng/mL rhTRAIL for an additional 4h. In parallel, some samples were exposed to hyperthermia at 42°C for 1, 2, 3, or 4h and then returned to 37°C for the remainder of the incubation period, respectively. Whole-cell extracts were analyzed with an immunoblotting assay using indicated antibodies.

To further evaluate cellular mechanisms during hyperthermia-based multimodal treatment-induced cytotoxicity and apoptosis, we examined proteins potentially involved in hyperthermia-generated cellular stress. We investigated the role of heat shock proteins, HSP70 and HSP90, as well as initiator caspase-2, in response to heat treatment alone and in combination with ART and rhTRAIL. To evaluate the significant impact of heat during multimodal treatment-induced apoptosis, we treated with lower doses of rhTRAIL and ART, and hyperthermia was applied for 1, 2, 3, or 4h. Western blotting results demonstrate increased PARP-1 cleavage and caspase cleavage (activation) with the addition of heat to ART + rhTRAIL, particularly with longer heating times (Figure 2). Caspase-2 showed a marked decrease in full-length protein (48 kDa) across heated multimodal-treated samples, indicating caspase-2 activation (Figure 2). Similarly, HSP70 expression increased in heat alone, heat + rhTRAIL, and multimodal treatment conditions compared to the control, and HSP90 showed the strongest expression during heat-involved multimodal treatment (Figure 2).

### Hyperthermia-based multimodal therapy significantly enhanced apoptosis, as confirmed by Annexin V and TUNEL immunofluorescence assays

**Figure 3.**
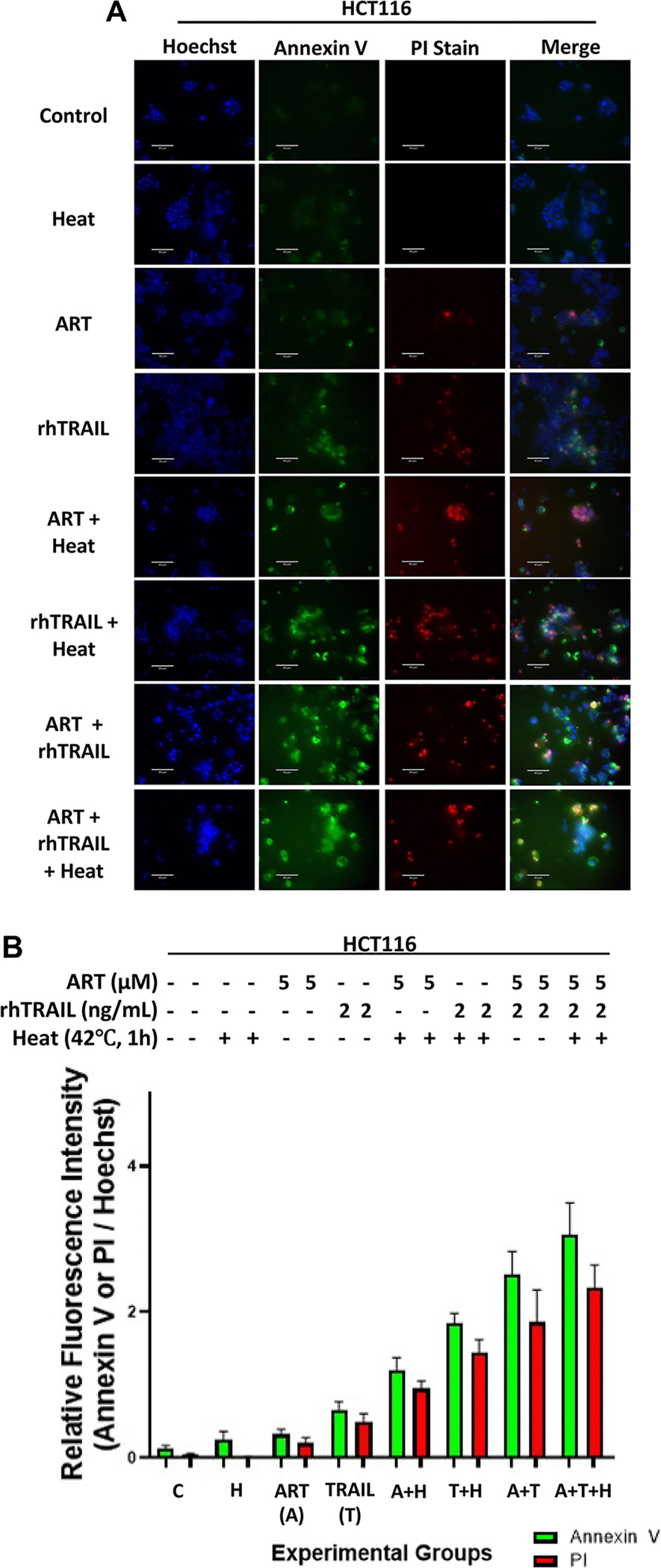

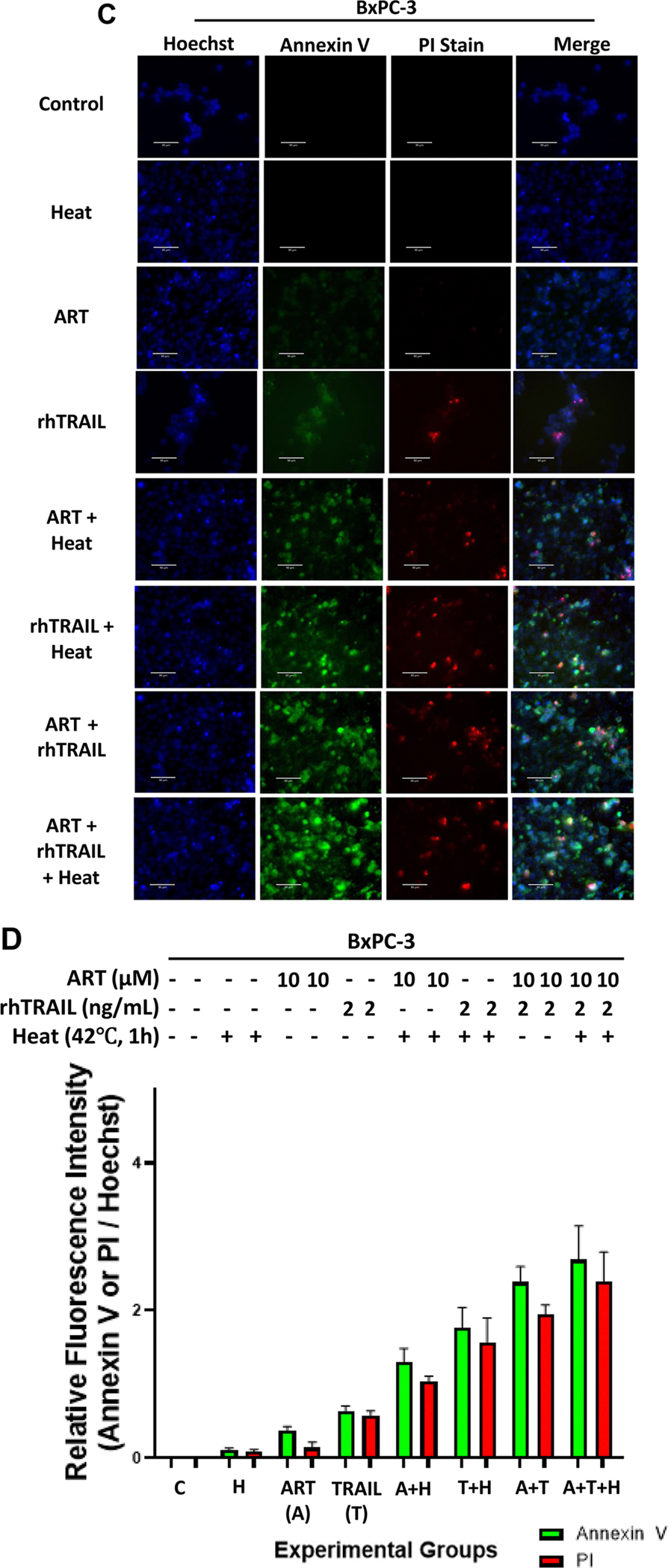

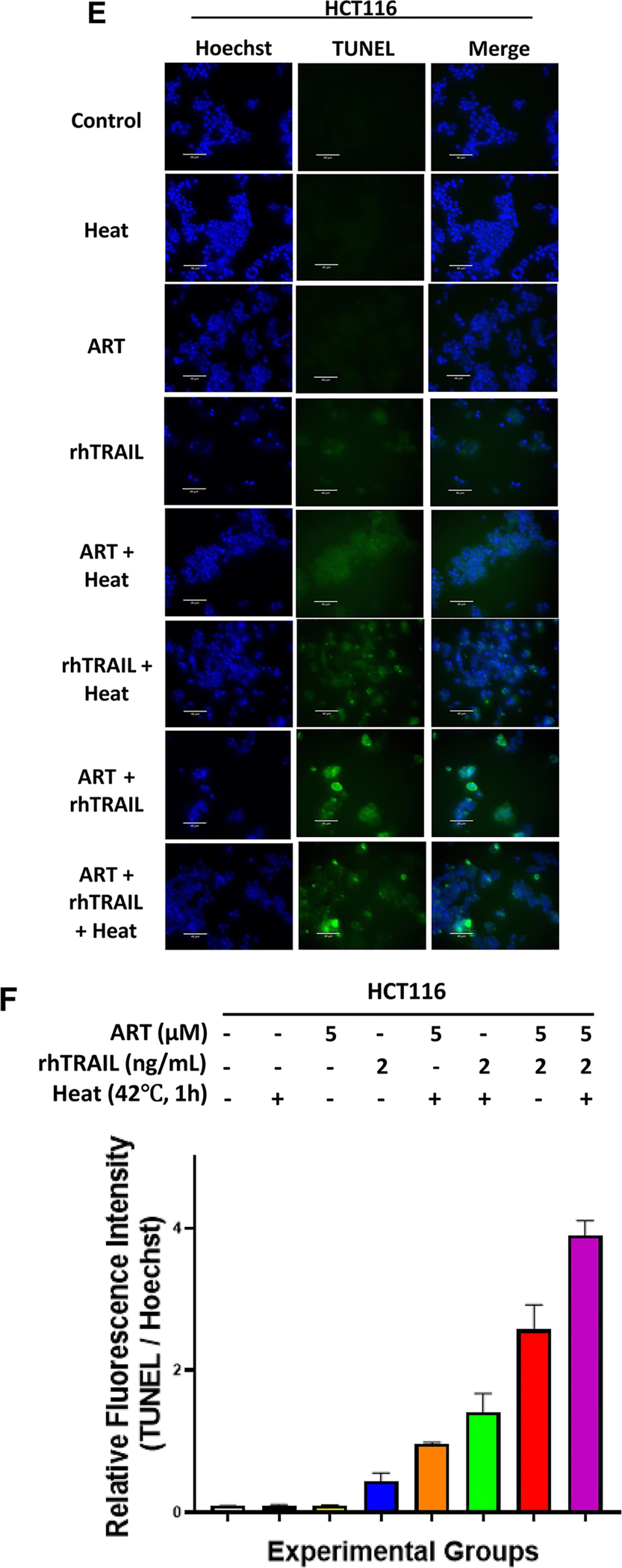
Immunofluorescence assays confirm that multimodal treatment enhances apoptosis in HCT116 and BxPC-3 cell lines. HCT116 and BxPC-3 cells were treated with 5μM and 10μM ART, respectively, for 20h at 37°C, followed by treatment with or without 2 ng/mL rhTRAIL for an additional 4h. Some samples were exposed to hyperthermia at 42°C for 1 h, then incubated at 37°C for the remaining 3h. (A, C) Annexin V staining assay was immediately performed after multimodal treatment was finished by staining cells with Annexin V-FITC (green), propidium iodide (PI) (red), and hoechst (blue), and analyzed using an ECHO fluorescence microscope. Representative images are shown (scale bar: 100μm) (B, D) ImageJ program was used to analyze the relative fluorescence intensity of each of the samples, measuring the hoechst stain intensity, Annexin V stain intensity (green channel), and PI stain intensity (red channel). The bar plot represents the ratio of Annexin V stain fluorescence intensity to hoechst stain fluorescence intensity, as well as PI stain intensity to hoechst stain fluorescence intensity, with error bars representing the mean ± SD from triplicate experiments. (E) TUNEL staining assay was performed on treated HCT116 cells and fluorescent images were examined under an ECHO fluorescence microscope. Representative images are shown (scale bar: 100μm). (F) ImageJ program was used to analyze the relative fluorescence intensity of each of the samples, measuring the hoechst stain intensity and the TUNEL stain intensity. The bar plot represents the ratio of TUNEL stain fluorescence intensity to hoechst stain fluorescence, with error bars representing the mean ± SD from triplicate experiments.

The apoptotic effectiveness of the multimodal treatment was additionally assessed using the Annexin V-FITC, which distinguishes stages of apoptosis: Annexin V-FITC staining (green) indicates early apoptosis, while propidium iodide (PI) staining (red) marks late apoptotic cells. In both HCT116 and BxPC-3 cells, minimal Annexin V and PI staining was observed in the untreated control and 42°C (1-h) groups, while ART or rhTRAIL alone induced modest staining compared to the pronounced Annexin V and PI staining observed in the combination treatments with heat (Figure 3(A–D)). These data confirm that multimodal treatment (ART + rhTRAIL + heat) markedly enhances apoptosis.

Subsequently, we performed the TUNEL assay (Terminal deoxynucleotidyl transferase dUTP nick-end labeling) to confirm DNA-fragmentation (a characteristic of apoptosis) in cells subjected to multimodal treatment (Figure 3(E,F)). The assay revealed increased FITC (green) fluorescence in the group treated with both ART and rhTRAIL, which was further enhanced by the addition of heat (Figure 3(E,F)). These findings consistently demonstrate that the multimodal treatment effectively promotes apoptosis.

**Figure 4.**
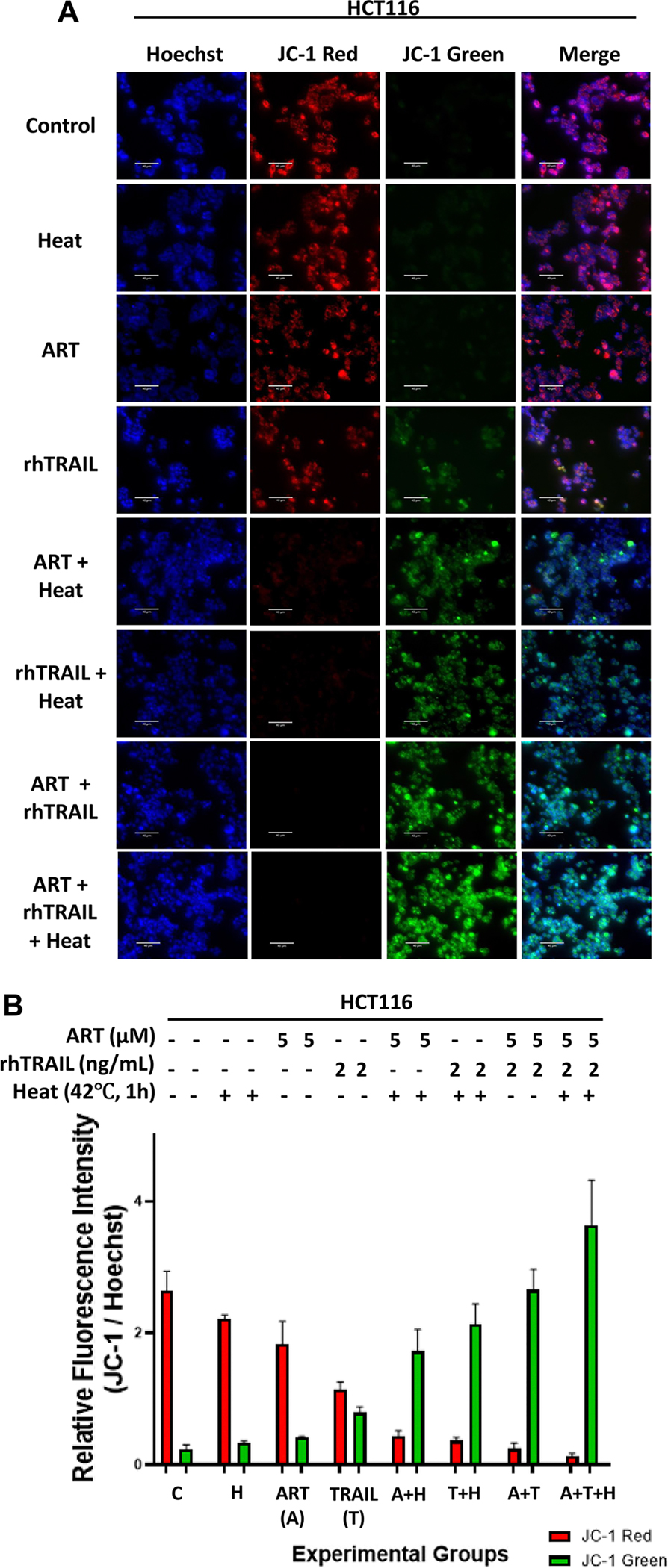
The multimodal treatment of ART + heat + rhTRAIL causes depolarization in the mitochondrial membrane potential. HCT116 cells were treated with 5μM of ART for 20h at 37°C and then with or without 2 ng/mL rhTRAIL for additional 4h. Some samples were subjected to hyperthermia treatment at 42°C for 1 h, and then incubated at 37°C for another 3h. (A) JC-1 staining assay was performed and results were analyzed using an ECHO fluorescence microscope. Representative images are shown (scale bar: 100μm). (B) ImageJ program was used to analyze the relative fluorescence intensity of each of the samples, measuring the hoechst stain intensity, JC-1 green intensity (green channel), and JC-1 red intensity (red channel). The bar plot represents the ratio of JC-1 green stain fluorescence intensity to hoechst stain fluorescence intensity, as well as JC-1 red stain intensity to hoechst stain fluorescence intensity, with error bars representing the mean ± SD from triplicate experiments.

Since mitochondria are key targets of apoptotic agents, we evaluated the impact of the multimodal treatment on mitochondrial function. We performed the JC-1 assay to evaluate changes in mitochondrial membrane potential during the multimodal treatment (Figure 4(A,B)). A decrease in red fluorescence and increase in green fluorescence was observed in samples treated with the combination of heat, ART, and rhTRAIL, indicating mitochondrial depolarization and stress associated with cytotoxic activity during the multimodal treatment [29] (Figure 4(A,B)).

### BID acts as a critical gatekeeper in the apoptotic pathway activated by hyperthermia-enhanced combination therapy with ART and rhTRAIL

**Figure 5.**
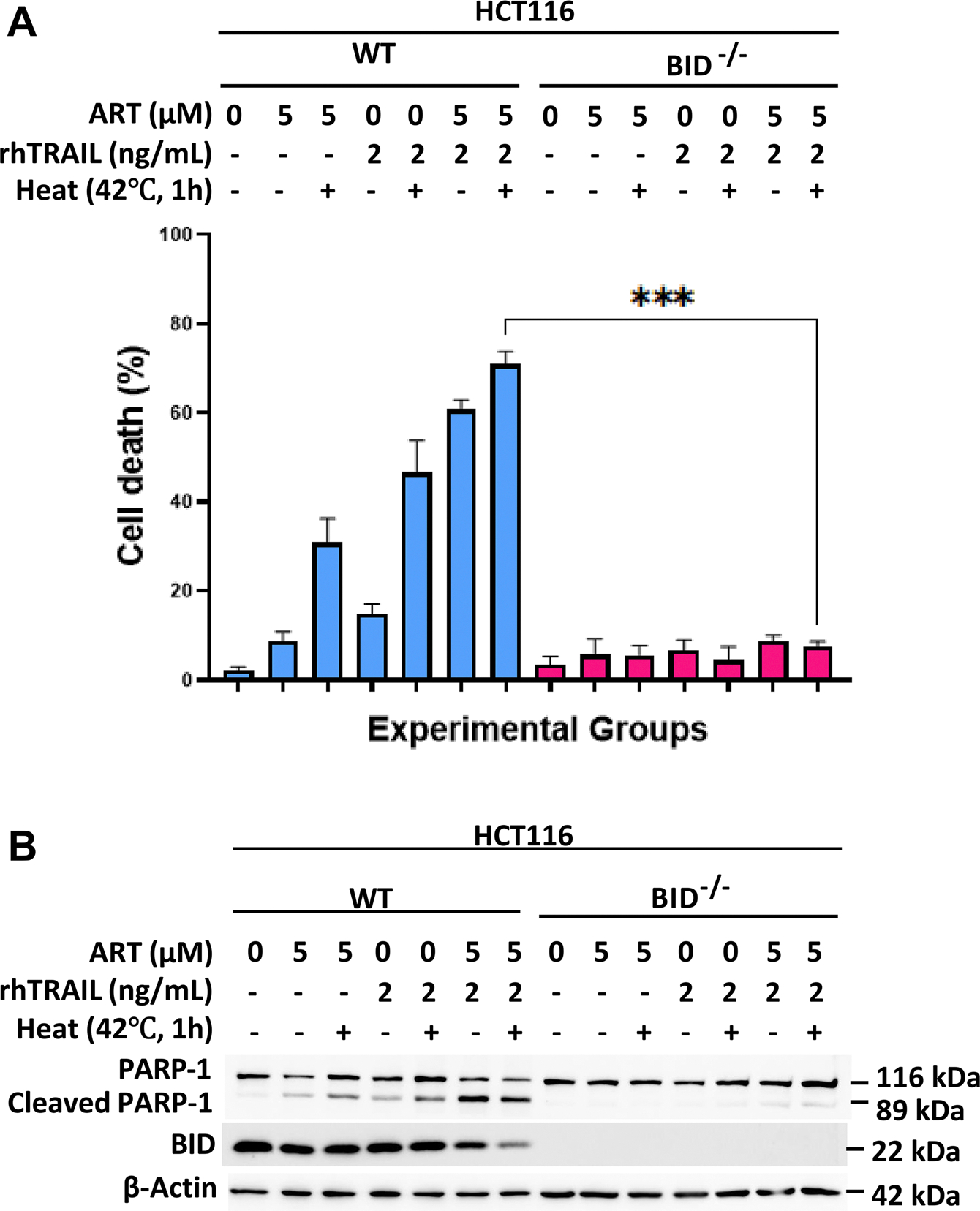
Role of BID in the synergistic multimodal treatment-induced apoptosis. HCT116 wild type and BID^−/−^ cells were treated with 5μM of ART for 20h at 37°C and then with or without 2 ng/mL rhTRAIL for additional 4h. Some samples were subjected to hyperthermia treatment at 42°C for 1 h, and then incubated at 37°C for another 3h. (A) Immediately following treatment, cell viability was evaluated using the trypan blue exclusion assay. Error bars represent the mean ± SD from triplicate experiments. P-values: ****, 0.0001. (B) Whole-cell extracts were analyzed with an immunoblotting assay using indicated antibodies.

**Figure 6.**
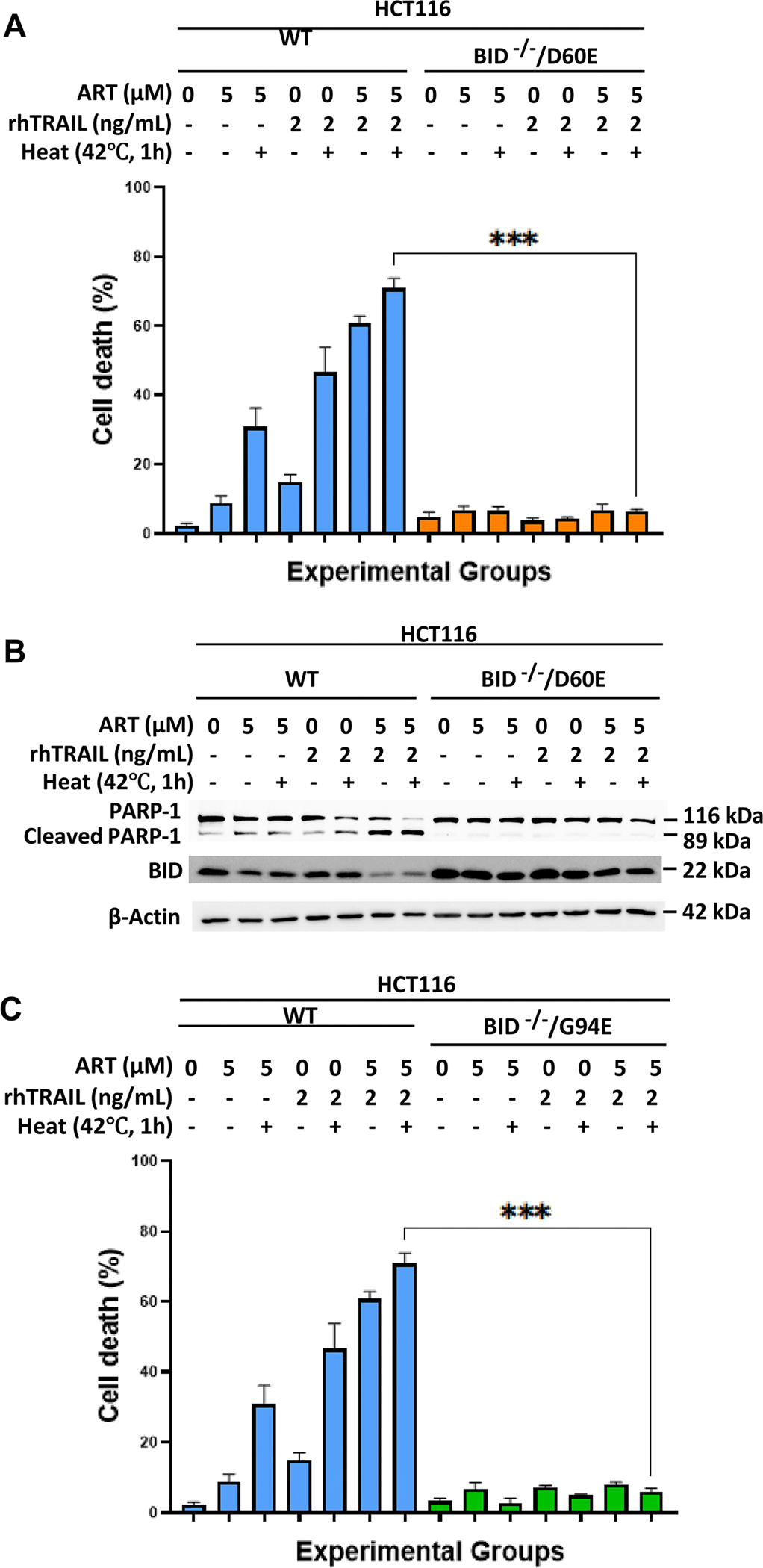

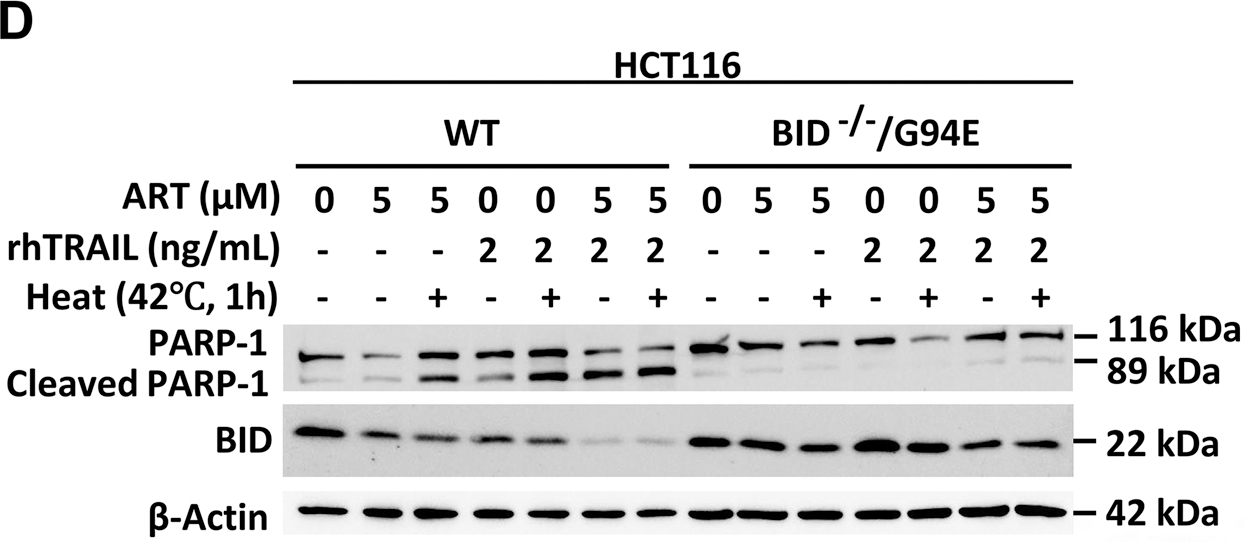
Functional BID is required for the synergistic multimodal treatment-induced apoptosis. HCT116 wild type, BID^−/−^/D60E, and BID^−/−^/G94E cells were treated with 5μM of ART for 20h at 37°C and then with or without 2 ng/mL rhTRAIL for additional 4h. Some samples were subjected to hyperthermia treatment at 42°C for 1 h, and then incubated at 37°C for another 3h. (A, C) Immediately after treatment, cell death was determined by a trypan blue exclusion assay. Error bars represent the mean ± SD from triplicate experiments. P-values: ***, 0.001. (B, D) To compare treatment effects in wild type HCT116 cells, BID^−/−^/D60E, BID^−/−^/G94E respectively, whole-cell extracts were analyzed with an immunoblotting assay.

Our previous research demonstrated that ART enhanced rhTRAIL-induced apoptosis relies on functional BID [15]. BID, a member of the Bcl-2 protein family, facilitates apoptosis by oligomerizing Bax and Bak proteins, which permeabilize the mitochondrial outer membrane, leading to caspase-9 and 3 activation, ending in apoptosis. To investigate whether hyperthermia-induced apoptosis in ART and rhTRAIL-treated cells depends on BID, we treated wild-type, BID-deficient (BID^−/−^/), and BID mutant (BID^−/−^/D60E or BID^−/−^/G94E) HCT116 cells with ART, rhTRAIL, and hyperthermia, both alone and in combination (Figures 5 and 6). The cell death assay using trypan blue stain revealed that in BID-deficient and BID type mutant cells, treatments with ART, rhTRAIL, and hyperthermia failed to induce cytotoxicity (Figures 5(A), 6(A,C)). Western blot analysis of PARP-1 cleavage confirmed that apoptosis was blocked in the BID deficient and BID mutant cell lines (Figures 5(B), 6(B,D)).

### Multimodal treatment-induced apoptosis is dependent on bax

**Figure 7.**
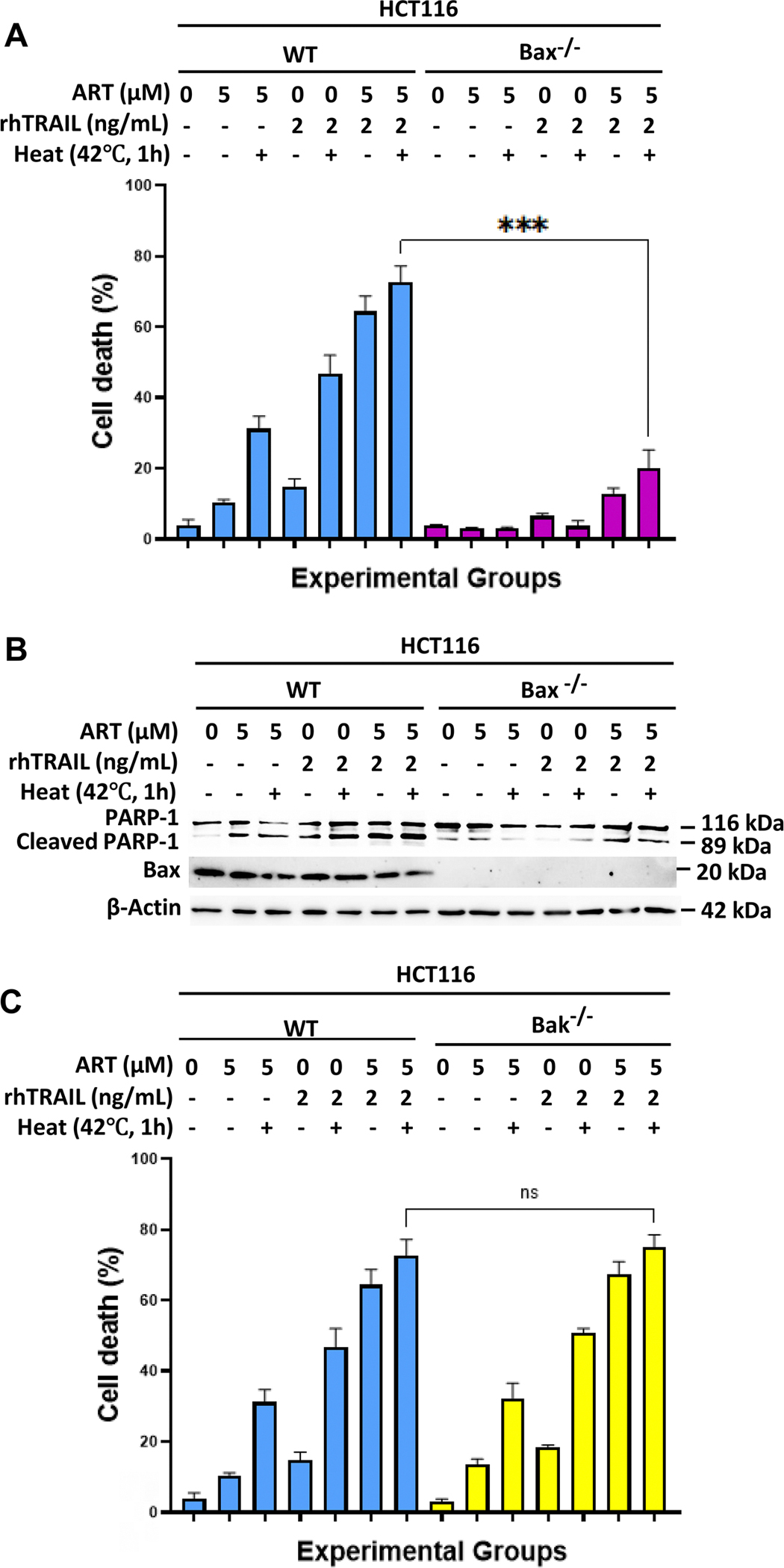

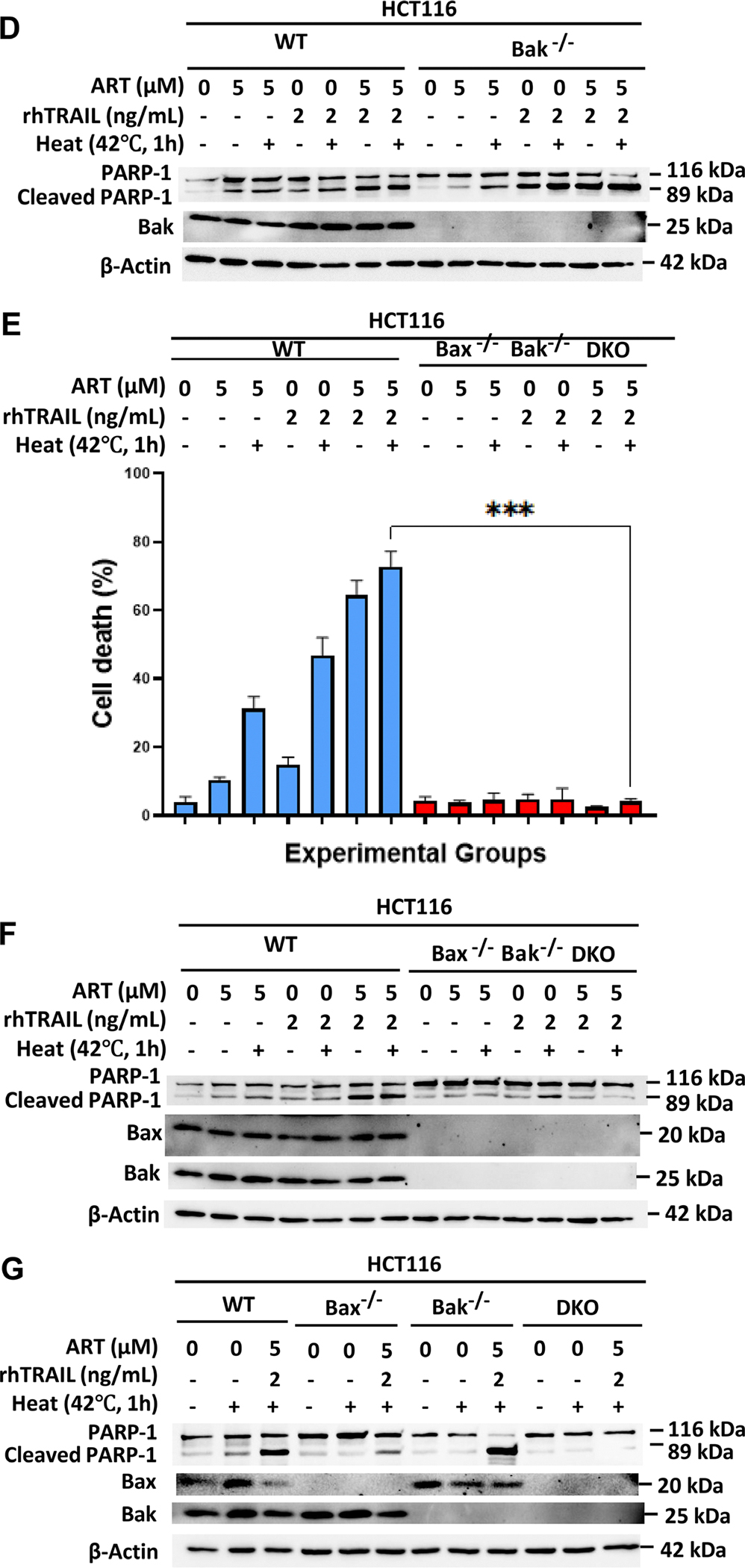
Role of Bax and Bak during multimodal treatment-induced apoptosis. HCT116 wild type, Bak ^−/−^, Bax ^−/−^, and Bak^−/−^/Bax^−/−^-DKO cells were treated with 5μM of ART for 20h at 37°C, and then with or without 2 ng/mL rhTRAIL for additional 4h. Some samples were subjected to hyperthermia treatment at 42°C for 1 h, and then incubated at 37°C for another 3h. (A, C, E) Immediately after treatment, cell death was determined by a trypan blue exclusion assay. Error bars represent the mean ± SD from triplicate experiments. P-values: ***, 0.001. n.s., not significant. (B, D, E, F) Whole-cell extracts were analyzed with an immunoblotting assay using indicated antibodies to compare treatment effects in wild-type HCT116 cells, Bax ^−/−^ HCT116 cells, Bak ^−/−^ HCT116 cells, and Bak^−/−^/Bax^−/−^-DKO HCT116 cells, respectively.

Similar to the role of BID, Bcl-2 protein Bax is essential for the increased apoptosis observed when ART and rhTRAIL are combined [23]. Bak, which is Bax’s counterpart protein, interestingly, has proven to not play an important role in the synergistic interactions [23]. We investigated whether Bax or Bak are critical in the hyperthermia-enhanced multimodal treatment by using Bax-deficient (Bax^−/−^), Bak-deficient (Bak^−/−^), and Bax/Bak double knockout (Bax^−/−^ Bak^−/−^ DKO) HCT116 cell lines (Figure 7). Cell viability assays and Western blotting for PARP-1 cleavage showed that Bax-deficient and double knockout cells had reduced apoptosis in the hyperthermia-enhanced treatment, with low cell death rates and minimal PARP-1 cleavage (Figure 7(A,B,E,F)). However, unlike Bax-deficient cells, Bak-deficient cells exhibited apoptosis levels comparable to wild-type cells, indicating that Bax, but not Bak, is essential in the hyperthermia-enhanced multimodal treatment-induced apoptosis (Figure 7(C,D)). Our observations were confirmed in Figure 7(G). Notably, heat alone has no significant effect as there is no PARP-1 cleavage shown (Figure 7(G)).

### Schematic representation of key observations

**Figure 8.**
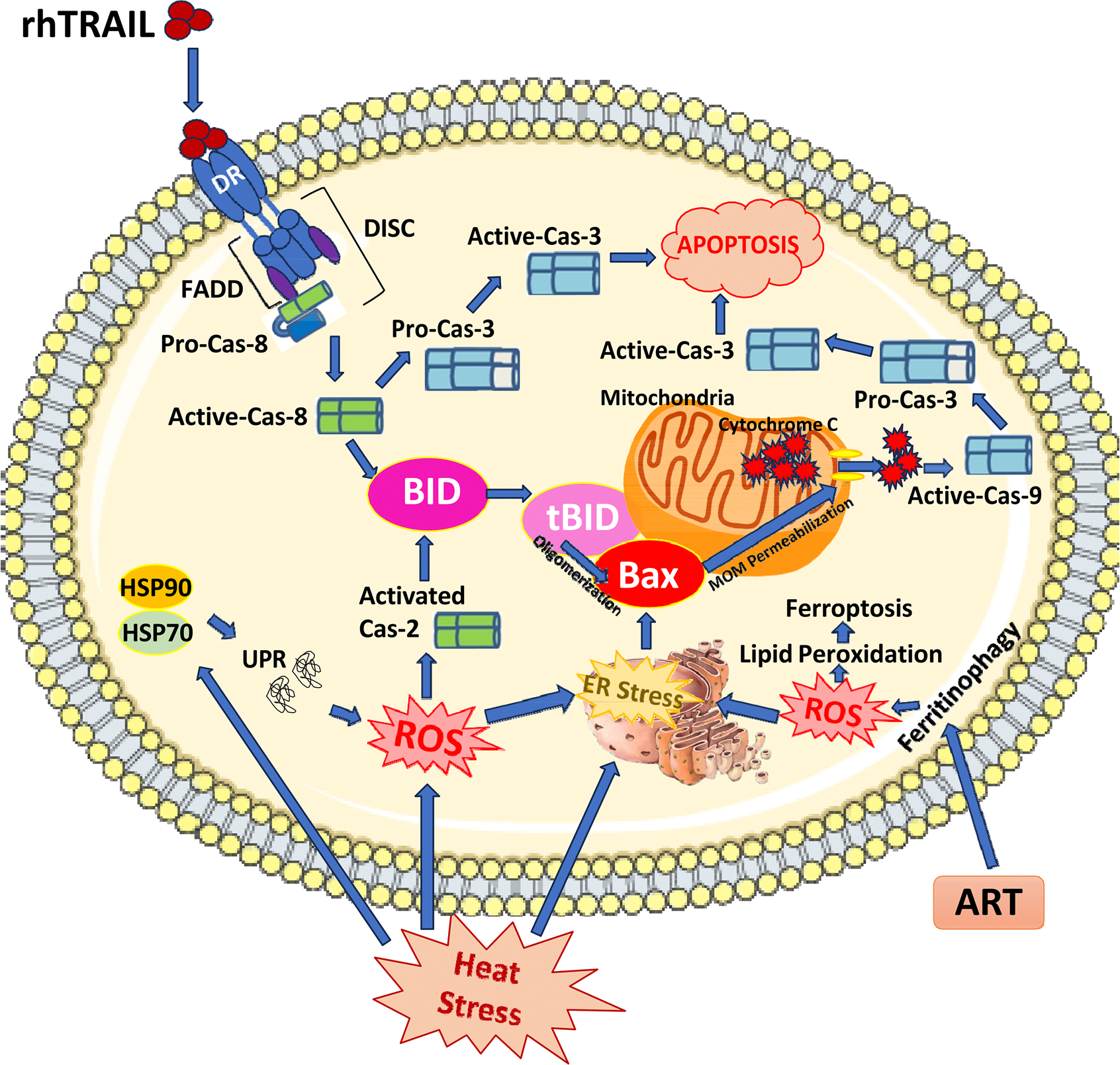
Diagram illustrating BID as the gatekeeper molecule in the hyperthermia enhanced multimodal therapy utilizing ART and rhTRAIL.

Figure 8 illustrates an overview diagram summarizing the experimental observations. Cytokine rhTRAIL induces apoptosis by selectively binding to DR4 and DR5 on cancer cells and initiates the caspase-activation cascade, leading to the truncation of BID. Truncated Bid (tBID) oligomerizes Bax, which permeabilizes the MOM releasing cytochrome c and activating initiator caspase-9. Caspase-9 activates caspase-3, and then active caspase-3 cleaves target proteins, including PARP-1, leading to cellular apoptosis. The anti-malarial drug, artesunate (ART), acts as a ferroptotic agent, causing ferritinophagy, generating ROS and inducing iron-dependent lipid peroxidation. ART also inhibits glutathione S-transferase, leading to reduced glutathione levels and activation of the endoplasmic reticulum (ER) stress response [13], which in turn promotes mitochondrial-dependent apoptosis through a BID-Bax-dependent pathway. Mild hyperthermia induces heat stress in the cell, leading to the generation of ROS and upregulation of heat shock protective proteins, HSP70 and HSP90. Under mild hyperthermic conditions, HSP70 and HSP90 work to prevent the Unfolded Protein Response (UPR). However, heat stress in combination with ART-enhanced, rhTRAIL-mediated apoptosis may overwhelm HSP capacity, impairing proteostasis and lead to the UPR and ER stress responses. Additionally, the generation of ROS from hyperthermia has been suggested to lead to small levels of initiator-caspase-2 activation, which is significantly increased during hyperthermia-based multimodal treatment. The diagram illustrates hyperthermia-enhanced ART and rhTRAIL-induced apoptosis through ROS production and crosstalk between the ER-stress-mediated mitochondrial pathway and the BID-Bax-mitochondrial apoptotic pathway.

## Discussion

This study investigated the potential of a hyperthermia-based multimodal therapy, combining ART and rhTRAIL, to enhance apoptosis in HCT116 colorectal carcinoma cells and pancreatic adenocarcinoma BxPC-3 cells. In cells treated with hyperthermia-based multimodal treatment, our results demonstrate change in cell morphology, increase in cytotoxicity, caspase-targeted proteins indicative of apoptosis, Annexin V/PI positivity, TUNEL labeling of DNA fragmentation, and JC-1 mitochondrial membrane depolarization (Figures 1–4). Using BID and Bax-deficient cell lines, we found that hyperthermia significantly potentiates ART and rhTRAIL-induced apoptosis through the BID-Bax-dependent mitochondrial pathway. These results are consistent with previous reports showing that heat enhances rhTRAIL efficacy through ROS generation, caspase activation, and mitochondrial dysfunction [9,24].

In the present study, mild hyperthermia (42°C for 1 h) alone does not induce apoptosis (Figure 1). Although heat shock proteins such as HSP70 and HSP90 are upregulated under mild hyperthermic conditions (Figure 2) to regulate protein folding, prevent denaturation, and promote cellular homeostasis and thermal tolerance [30,31], the combination of mild hyperthermia with the ferroptotic agent ART, the apoptotic agent rhTRAIL, or both, leads to enhanced apoptosis. These findings suggest that ART-enhanced, rhTRAIL-induced apoptosis causes cellular damage that exceeds the protective capacity of HSP70 and HSP90. Conceivably, from our results, extended heat stress in combination with rhTRAIL-induced-apoptotic and ART-induced-ferroptotic signals could create an imbalance between the cellular need for HSPs and their available capacity to perform crucial roles in maintaining proteostasis [9,24,27,28,30,32]. Theoretically, if the protective functions of HSPs are overwhelmed by ART-and rhTRAIL-induced cytotoxicity, our results suggest cells undergo apoptosis induced by hyperthermia-enhanced, ART-rhTRAIL-combination treatment. Hypothetically, the disruption of HSPs activates the unfolded protein response (UPR) and ER stress pathways, including CHOP and ATF4, which are also upregulated by ART treatment [9,11,24]. ART additionally promotes ROS generation and impairs redox homeostasis via the ferroptosis pathway [13], contributing to mitochondrial dysfunction and sensitizing cells to apoptosis. Similarly, as established, rhTRAIL initiates the extrinsic cell death pathway, and via caspase-8, promotes BID activation, leading to the intrinsic-BID-Bax-mitochondrial-dependent-apoptosis pathway [9,13–17,33,34]. In addition, initiator caspase-2 has been implicated in small levels of cleavage of BID under conditions of heat stress and upregulated ROS, thereby serving as a critical upstream mediator linking hyperthermia to the BID-Bax-mitochondrial pathway of apoptosis [32–35]. Results in Figure 2 exhibit caspase-2 activation during hyperthermia-based multimodal treatment. Mechanistically, upon activation, caspase-2 cleaves BID into its truncated form, tBID, which translocates to the mitochondria where it promotes mitochondrial outer membrane permeabilization (MOMP) [33,34] (Figure 8). This event facilitates the release of pro-apoptotic factors such as cytochrome c into the cytosol, leading to apoptosome formation and activation of downstream caspases (caspase-9 and caspase-3), ultimately driving cell death [9,33,34]. However, even though caspase-2 has the molecular ability to cleave BID into tBID, it has been found to be less active than other initiator caspases, such as caspase-8 [33]. This implies that even though heat alone may induce some stress signals leading to caspase-2 activation, it may not produce enough active tBID alone to cause widespread apoptosis. The results reflect this hypothesis, where little PARP-1 cleavage is observed after heat treatment alone, as well as little signals of cytotoxicity (Figure 1(A–C)). These findings may elucidate why the incorporation of hyperthermia with additional apoptotic and ferroptotic agents markedly augments apoptosis, relative to hyperthermia alone. This suggests a synergistic crosstalk between hyperthermia, ferroptotic, and apoptotic pathways, with BID at the center.

Our data support this mechanism of BID as the gatekeeper of synergistic-apoptosis induced by heat, ART, and rhTRAIL. Importantly, BID-deficient or mutant cells failed to undergo apoptosis under the combined treatment, underscoring BID’s role as a key mediator in multimodal-induced apoptosis (Figures 5 and 6). Our data also revealed that Bax, but not Bak, is essential for the observed cytotoxic effects, consistent with prior findings that link Bax to ART and rhTRAIL-induced mitochondrial apoptosis [23] (Figure 7). As established, tBID translocates to mitochondria, facilitating Bax activation, MOMP, and downstream caspase signaling [9,15,33,34]. ROS and ER stress further enhance this process by impairing anti-apoptotic Bcl-2 proteins or lowering the threshold for Bax activation [13,36]. These findings demonstrate that hyperthermia-enhanced multimodal treatment is dependent on functional BID and Bax (Figure 8). Therefore, a better understanding of the BID-Bax integration pathway has translational potential as it could guide patient grouping and prioritization in clinical trials, especially for patients diagnosed with CPC. Patients possessing tumors with higher expression of BID and Bax proteins may be more responsive to therapies dependent on BID-Bax-mitochondrial-dependent apoptosis. For example, in clinical practice, pretreatment tumor biopsies could be screened for BID-Bax protein expression in order to identify CPC patients that would likely respond to the multimodal therapy.

Conversely, patients with BID-Bax-deficient tumors may require alternative therapeutic strategies, such as the use of other apoptosis-inducing agents that do not rely on the BID-Bax-pathway. We have preliminary data on a drug called DZ-1-ART which induces mitochondria associated apoptosis independent of the BID-Bax-axis. DZ-1-ART is the conjugated form of DZ-1 and artesunate (ART). DZ-1 is a type of heptamethine cyanine dye, which can target the over-expression of organic anion transporting polypeptides (OATPs) in cancer cells for specific selectivity [37]. When DZ-1 is conjugated to ART, we have observed cytotoxic cell death and apoptosis in multiple types of cancer cell lines (unpublished data). This drug is just one example of an option for treatment in patients with BID/Bax mutations or that have tumors with low BID-Bax protein expression. Future studies should explore if drugs like DZ-1-ART or other BID-Bax dependent therapeutics can be enhanced by the addition of heat. Continually, strategies to overcome resistance in BID-Bax-deficient tumors, such as approaches aimed at restoring the functionality of the BID-Bax-axis in tumors, upregulating BID-Bax protein expression in patient tumor cells, or alternative combination regimens that bypass this dependency can be emphasized.

In summary, the present study reveals that BID acts as a key gatekeeper molecule of apoptosis during hyperthermia-based multimodal treatment (Figure 8). We hypothesize that hyperthermia enhances the apoptotic and ferroptotic effects of ART and rhTRAIL by promoting oxidative and ER stress, thereby sensitizing cells and amplifying signals in both the intrinsic BID-Bax-mitochondrial axis, and extrinsic-deat h-receptor-mediated apoptotic pathway. This crosstalk provides a mechanistic rationale for targeting CPC tumors with intact BID-Bax signaling during our proposed hyperthermia-based multimodal treatment, potentially improving patient selection and CPC treatment outcomes.

## Figures and Tables

**Table 1.** One-way ANOVA statistical analysis, P-values for ART, rhTRAIL, and hyperthermia.

Drug Comparison	Statistical Significance

Heat vs 5 μM ART + Heat	***
Heat vs 10 μM ART + Heat	***
Heat vs 1 ng/mL rhTRAIL + Heat	***
Heat vs 2 ng/mL rhTRAIL + Heat	***
Heat vs 5 μM ART + 1 ng/mL rhTRAIL + Heat	***
Heat vs 5 μM ART + 2 ng/mL rhTRAIL + Heat	***
Heat vs 10 μM ART + 1 ng/mL rhTRAIL + Heat	***
Heat vs 10 μM ART + 2 ng/mL rhTRAIL + Heat	***
5 μM ART vs 5 μM ART + Heat	**
10 μM ART vs 10 μM ART + Heat	***
1 ng/mL rhTRAIL vs 1 ng/mL rhTRAIL + Heat	***
2 ng/mL rhTRAIL vs 2 ng/mL rhTRAIL + Heat	***
5 μM ART + 1 ng/mL rhTRAIL vs 5 μM + 1 ng/mL rhTRAIL + Heat	*
5 μM ART + 2 ng/mL rhTRAIL vs 5 μM + 2 ng/mL rhTRAIL + Heat	*
10 μM ART + 1 ng/mL rhTRAIL vs 10 μM + 1 ng/mL rhTRAIL + Heat	**
10 μM ART + 2 ng/mL rhTRAIL vs 10 μM + 2 ng/mL rhTRAIL + Heat	*

**Table 2.** Combination Index data for ART, rhTRAIL and hyperthermia.

Drug Combination	Combination Index (CI)	Effect

5 μM ART + Heat	0.72082	Moderate Synergy
10 μM ART + Heat	0.67044	Moderate Synergy
1 ng/mL rhTRAIL + Heat	0.69660	Moderate Synergy
2 ng/mL rhTRAIL + Heat	0.67728	Moderate Synergy
5 μM ART + 1 ng/mL rhTRAIL	0.00386	Strong Synergy
5 μM ART + 2 ng/mL rhTRAIL	0.00651	Strong Synergy
10 μM ART + 1 ng/mL rhTRAIL	0.00149	Strong Synergy
10 μM ART + 2 ng/mL rhTRAIL	0.00127	Strong Synergy
5 μM + 1 ng/mL rhTRAIL + Heat	0.57410	Moderate Synergy
5 μM + 2 ng/mL rhTRAIL + Heat	0.56592	Moderate Synergy
10 μM + 1 ng/mL rhTRAIL + Heat	0.52348	Moderate Synergy
10 μM + 2 ng/mL rhTRAIL + Heat	0.50462	Moderate Synergy

## Data Availability

Research materials that were generated in the studies including plasmid DNA constructs will be made freely available to the scientific research community as soon as this manuscript has been documented in a publication. Raw data were generated at the Cedars-Sinai Medical Center. Derived data supporting the findings of this study are available from the corresponding author Dr. Yong J. Lee on request.
